# Major alteration in coxsackievirus B3 genomic RNA structure distinguishes a virulent strain from an avirulent strain

**DOI:** 10.1093/nar/gku706

**Published:** 2014-07-29

**Authors:** Jerome Prusa, Johanna Missak, Jeff Kittrell, John J. Evans, William E. Tapprich

**Affiliations:** 1 Biology Department, University of Nebraska at Omaha, Omaha, NE 68182, USA; 2 Department of Family Medicine, University of Nebraska Medical Center, Omaha, NE 68198, USA; 3 Genetics, Cell Biology and Anatomy, University of Nebraska Medical Center, Omaha, NE 68198, USA; 4 Department of Pathology, University of Colorado Anshutz Medical Campus, Denver, CO 80045, USA

## Abstract

Coxsackievirus B3 (CV-B3) is a cardiovirulent enterovirus that utilizes a 5′ untranslated region (5′UTR) to complete critical viral processes. Here, we directly compared the structure of a 5′UTR from a virulent strain with that of a naturally occurring avirulent strain. Using chemical probing analysis, we identified a structural difference between the two 5′UTRs in the highly substituted stem-loop II region (SLII). For the remainder of the 5′UTR, we observed conserved structure. Comparative sequence analysis of 170 closely related enteroviruses revealed that the SLII region lacks conservation. To investigate independent folding and function, two chimeric CV-B3 strains were created by exchanging nucleotides 104–184 and repeating the 5′UTR structural analysis. Neither the parent SLII nor the remaining domains of the background 5′UTR were structurally altered by the exchange, supporting an independent mechanism of folding and function. We show that the attenuated 5′UTR lacks structure in the SLII cardiovirulence determinant.

## INTRODUCTION

Coxsackievirus B3 (CV-B3) is an agent of serious human diseases including myocarditis and cardiomyopathy (1–4). This virus is a member of the *Picornaviridae* family (Order *Picornavirales*, Family *Picornaviridae*, Genus *Enterovirus*, species *Human enterovirus B*, type: CV-B3) (5). Similar to other enteroviruses, including the well characterized and closely related poliovirus (PV), the CV-B3 genome is commonly organized into four major regions: a 742 nucleotide 5′untranslated region (5′UTR), a single open reading frame encoding a 2185 amino acid polypeptide, a 98 nucleotide 3′UTR and a poly(A) tail (6–8). A 22 amino acid protein called VPg is covalently linked to the 5′ end of the enteroviral genome (9,10). The structure and function of the picornavirus 5′UTR has been studied extensively because it plays a critical role in all stages of the viral infection cycle.

The 5′UTR of CV-B3 and other enteroviruses can be divided into seven highly structured RNA domains (11) that are generally divided into two functionally distinct regions (12–15). One functional region is a highly conserved 5′ terminal cloverleaf that encompasses domain I as well as an adjacent pyrimidine rich single stranded sequence. The cloverleaf and pyrimidine rich region are required for positive and negative strand synthesis during viral genome replication (14–19). The second functional region is composed of domains II–VII and is collectively called the internal ribosomal entry site (IRES). The CV-B3 and PV IRESs are considered type 1 and are structurally distinct from the type 2 IRESs, best characterized in encephalomyocarditis virus and foot-and-mouth disease virus (20,21), or the type 3 IRESs found in Hepatitis A virus (22). Irrespective of type, the IRES is responsible for initiating cap-independent translation in picornaviruses (21–23). A large body of evidence shows that the cloverleaf and the IRES derive their functionality from both primary and higher order RNA structures (24–26). Previous work with PV also supports a synergistic interplay between these two major functional regions (27–29). However, a structure-based mechanism describing how these interactions mediate virus multiplication and virulence remains enigmatic.

Several virulence determinants have been identified in the enterovirus genome including regions encoding structural and non-structural proteins such as the capsid proteins VP1–VP4 and the non-structural viral polymerase 3D (30–32). In addition to coding regions, virulence determinants have been identified in the 5′UTR of CV-B3 as well as other clinically important and well-studied enteroviruses. For example, PV, coxsackievirus B1 and human enterovirus 71 all have virulence determinants localized in the 5′UTR (33–40). The most famous and perhaps best-studied example of 5′UTR virulence determinants are nucleotide positions 472, 480 and 481 in domain V of the PV 5′UTR that are responsible for the Sabin polio vaccine strains (41–43). A single nucleotide cardiovirulence determinant in the CV-B3 5′UTR at position 234 in domain III has been previously reported (44,45).

The region connecting domain I and domain II combined with domain II as shown in the most recent structural model for the CV-B3 5′UTR (11) has been commonly referred to as stem loop II (SLII, positions 88–181). SLII has been identified as a virulence determinant in multiple enteroviruses including CV-B3 (35,37). Chimeric constructs with SLII exchanged between virulent and avirulent strains were used to characterize SLII as a virulence determinant. Dunn *et al.* exchanged SLII (88–181) between the naturally occurring avirulent CV-B3/CO and virulent CV-B3/AS strains and showed that cardiovirulence in a murine model follows SLII of CV-B3/AS (37). Similar results were observed when the SLII in a non-cardiovirulent echovirus 12 (ECV12) was exchanged with SLII of a full-length infectious clone of CV-B3. Once again, cardiovirulence followed SLII of the infectious CV-B3 clone in all the chimeric constructs tested (35). Additional studies examining different enterovirus species confirm the role of SLII in virulence (33–40). The present study investigates a structural mechanism underlying SLII-dependent cardiovirulence.

The generally accepted mechanism for 5′UTR-dependent virulence attenuation is a mutation or accumulation of mutations that change critical RNA secondary and tertiary structures. Such structural alterations mediate inefficiencies in viral processes such as genome replication and cap-independent translation. This structure-based mechanism of virulence attenuation has motivated efforts to solve and compare virulent and avirulent 5′UTR structures (46–49). Most of these comparisons are made using theoretical approaches, most notably RNA folding algorithms and sequence comparison analysis. These theoretical approaches are useful and have repeatedly predicted secondary structure differences in virulent and avirulent 5′UTR regions where the primary sequences are not conserved, including those in the CV-B3 SLII (34,35,49). These theoretical approaches have further strengthened the model suggesting that structural alterations in SLII underlie changes in CV-B3 virulence phenotypes.

Despite the important contributions made by the theoretical approaches, experimental methods are needed to solve 5′UTR structures. In this study, chemical probing techniques are employed to show a drastic secondary structure shift in the SLII of the naturally occurring avirulent strain, CV-B3/GA. These results are in agreement with previous reports showing that SLII is an enterovirus virulence determinant, but add substantially to the structural underpinnings of that functional role (28,33–38). The chemical probing data show that the avirulent CV-B3/GA SLII is unstructured in contrast to the highly structured SLII in virulent CV-B3/28.

## MATERIALS AND METHODS

### Viral plasmids

Full-length CV-B3/28 and CV-B3/GA genomic constructs were engineered and kindly provided by Dr Nora Chapman at the Enterovirus Research Laboratory, University of Nebraska Medical Center, Omaha, NE, USA. The domain II chimeras (28 SLII GA and GA SLII 28) were also engineered by Dr Chapman and created by exchanging nucleotides 104–184 in CV-B3/GA and CV-B3/28. The plasmids were modified with the inclusion of a 38 nucleotide ribozyme sequence (ATGAGGCCGAAAGGCCGAAAACCCGGTATCCCGGGTTC) upstream of the 5′UTR and a T7 promoter sequence (TAATACGACTCACTATAGGG) upstream of the ribozyme. Upon *in vitro* transcription from the T7 promoter, the ribozyme sequence self-cleaves to produce the authentic uridine at the 5′ end of the UTR (50). The cleaved transcript does not have VPg.

Plasmid DNA was isolated using Qiagen (Valencia, CA, USA) QIAprep Spin Miniprep Kit according to the manufacturer's protocol. The 5′UTR template was generated by linearizing the plasmids. Restriction enzyme *Ecl*136II was used to linearize the plasmids encoding CV-B3/28 5′UTR sequence (756 bp) and *Bss*HII was used to linearize the plasmids encoding the CV-B3/GA 5′UTR sequence (757 bp). The sticky ends created by the *Bss*HII digestion were filled in with Klenow. Digested DNA was purified by phenol and phenol/chloroform extraction. The digested DNA was precipitated by adding two volumes of 95% ethanol and incubating overnight at −20°C. The DNA was then pelleted, washed with 70% ethanol and resuspended with 21 μl of 10 mM Tris, 1 mM EDTA Buffer (TE) pH 7.6.

### Viral RNA transcription

*In vitro* transcriptions were done using a Megascript kit (Ambion, Austin, TX, USA) according the manufacturer's protocol. The transcription reactions used 4 μg of digested template DNA and the total reaction volume was brought to 80 μl. The transcription reactions were incubated for 4 h at 37°C. The DNA template was removed by adding 4 μl of DNase I provided in the Megascript kit and incubating for 30 min at 37°C. The transcription reactions were stopped with 460 μl of RNase free water and 60 μl of 0.5 M-ammonium acetate. The RNA was extracted with phenol-chloroform-isoamyl alcohol (25:24:1) and precipitated by adding 600 μl of isopropyl alcohol and incubating overnight at −20°C. The RNA was pelleted by centrifugation for 20 min at 4°C. The RNA was washed with 400 μl of 70% ethanol and resuspended with 80 μl of TE buffer, pH 7.6. The RNA was stored at −80°C.

### RNA structural analysis

#### DMS modifications

DMS modification reactions contained 15 μg of RNA in 100 μl of DMS/Kethoxal buffer solution (40 mM K-cacodylate [pH 7.2], 10 mM MgCl_2_, 50 mM NH_4_Cl). The RNA was denatured by incubating at 80°C for 2 min then slow cooled to 40°C to allow the RNA to fold into its native structure. The DMS modification solution was prepared by diluting 20% DMS with 95% ethanol to make the final DMS concentration 2%. The DMS modification solution (2 μl) was added to the RNA and incubated at 37°C for 10 min. The modification reaction was stopped by adding 25 μl of solution containing 1 M Tris-HCl (pH 7.5), 1 M β-mercaptoethanol, 0.1 M EDTA. Control RNA was subjected to the same steps in the protocol except 2 μl of DMS/Kethoxal buffer was added in place of the DMS modification solution.

#### Kethoxal modifications

Kethoxal modification reactions contained 15 μg of RNA in 100 μl of DMS/Kethoxal buffer solution (40 mM K-cacodylate [pH 7.2], 10 mM MgCl_2_, 50 mM NH_4_Cl). The RNA was denatured by incubating at 80°C for 2 min then slow cooled to 40°C to allow the RNA to fold into its native structure. Five microliter of 1.5 M Kethoxal was added to the RNA and the reaction was incubated at 37°C for 10 min. The Kethoxal modification reaction was stopped by adding 50 μl of a solution containing 150 mM sodium acetate, 250 mM potassium borate (pH 7.0). Control RNA was subjected to the same steps in the protocol except 5 μl of DMS/Kethoxal buffer was added in place of the Kethoxal modifying solution.

#### CMCT modifications

CMCT modification reactions contained 15 μg of RNA in 50 μl of CMCT buffer solution (40 mM K-borate [pH 8.0], 10 mM MgCl_2_, 50 mM NH_4_Cl). The RNA was denatured by incubating at 80°C for 2 min then slow cooled to 40°C to allow the RNA to fold into its native structure. The CMCT modification solution was prepared by dissolving solid CMCT with CMCT buffer solution for a final concentration of 42 mg/ml. Fifty microliter of the CMCT modification solution was added to the RNA and the reaction was incubated at 37°C for 10 min. The CMCT modification reaction was stopped with 300 μl of 95% ethanol. Control RNA was subjected to the same steps in the protocol except 50 μl of CMCT buffer was added in place of the CMCT modification solution.

All modified RNA was precipitated with ethanol and sodium acetate, pelleted, washed with 70% ethanol and resuspended with aqueous solution. The DMS- and CMCT-modified RNA was resuspended with TE solution (pH 7.6). The Kethoxal-modified RNA was resuspended with 25 mM potassium borate solution (pH 7.0).

#### Oligonucleotide labelling

The modified nucleotides were identified with reverse transcriptase (RT) primer extension analysis. A collection of oligonucleotide primers was spaced along the 5′UTR to provide full coverage. The oligonucleotide primers were 5′ end labelled with [^32^P] ATP and T4 polynucleotide kinase. The oligonucleotide labelling reactions contained 100 pmol of oligonucleotide DNA, 4 μl of 5X forward buffer (350 mM Tris-HCl [pH 7.6], 500 mM KCl, 50 mM MgCl_2_, 5 mM 2-mercaptoethanol), 100 μCi of [γ-^32^P]ATP (10 μCi/μl) and 10 units of T4 polynucleotide kinase. The total volume of the labelling reaction was 20 μl. The reaction was incubated at 37°C for 40 min followed by incubation at 60°C for 20 min to inactivate the kinase enzyme. Thirty microliter of TE (pH 7.6) was added to the labelling reaction to dilute the labelled oligonucleotides to 2 pmol/μl.

#### Annealing reaction

The annealing reaction contained 1 μg of modified RNA, 2 μl of 5X Annealing buffer (250 mM Tris-HCl [pH 8.3], 200 mM KCl) and 1 μl of ^32^P-labelled oligonucleotide for a total volume of 10 μl. The RNA was denatured by incubating at 80°C for 2 min then slow cooled to 40°C.

#### Extension reaction

The extension reaction contained 2 μl of the annealing reaction, 2 μl of 2X extension mix (100 mM Tris-HCl [pH 8.3], 80 mM KCl, 12 mM MgCl_2_, 4 mM of each of the four deoxynucleoside triphosphates [dATP, dCTP, dGTP, dTTP]) and 1 μl (1 unit/μl) of avian myeloblastosis virus (AMV) RT (Life Sciences, St Petersburg, FL, USA). Sequencing ladders were generated by adding primer annealed unmodified RNA to one of four different termination mixes (50 mM Tris-HCl [pH 8.3], 40 mM KCl, 6 mM MgCl_2_, 1 mM of each deoxynucleoside triphosphate [dATP, dCTP, dGTP, dTTP] and 0.1 mM of one dideoxynucleoside triphosphate [ddATP, ddCTP, ddGTP or ddUTP]) and 1 μl (1 unit/μl) of (AMV) RT. All the reaction mixtures were incubated at 40°C for 25 min. The extension reactions were stopped by adding 2 μl of solution containing 95% formamide, 20 mM EDTA, 0.05% bromophenol blue, 0.05% xylene cyanol. The reaction mixtures were stored at −80°C.

#### Gel electrophoresis

The primer extension reactions were analysed on 12% polyacrylamide sequencing gels. The extension products were electrophoresed through the sequencing gel at constant 60W for 3–6 h with TBE buffer (90 mM Tris-Borate [pH 8.3], 1 mM EDTA). Mobility through the sequencing gel was visualized and captured with a Packard Cyclone phosphorimager (Packard Instrument Company, Meriden, CT, USA).

#### Sequence comparison

Complete full-length genomes of 170 enteroviruses were obtained from http://www.ncbi.nlm.nih.gov/nucleotide/. The CV-B3/28 accession number is AY752944 (49). The CV-B3/GA accession number is AY673831 (49). The enteroviruses that were included in the comparison were chosen because their full-genome sequences are available in GenBank. Partial sequences were not included in the comparison. The sequences were aligned using Invitrogen Vector NTI Advance 11 (Life Technologies, Grand Island, NY, USA). The aligned sequences were used to generate a sequence logo using http://weblogo.berkeley.edu/logo.cgi.

## RESULTS

### Chemical probing analysis

The three chemical probes employed in this study modify positions on the nucleotide bases that are involved in Watson–Crick base pairing (51). Primarily, these chemicals and their modification specificity allow Watson–Crick base paired positions to be distinguished from non-Watson–Crick positions. DMS methylates the N1 position of adenosine and the N3 position of cytosine. CMCT methylates the N3 position of uridine and Kethoxal creates a cyclic adduct using positions N1 and N2 of guanosine. Bases are protected from modification when they are involved in Watson–Crick interactions or when they are located in a solvent inaccessible region of the folded molecule. Modified nucleotides are identified by RT primer extension reactions. During the RT extension reaction, modified positions will stop the extension. The labelled cDNA fragments produced from the extension reaction are separated on a 12% polyacrylamide gel. For interpreting the position of modification, bands in the sequencing tracks (A,C,G,U) migrate one position above bands in the experimental modification tracks due to addition of the dideoxynucleotide in the sequencing track as compared to RT stopping on the base prior to the DMS, CMCT or Kethoxal modified nucleotides. All chemical probing experiments were conducted at least three times on independent preparations of RNA.

### Structural comparison of SLII regions

**Figure 1.**
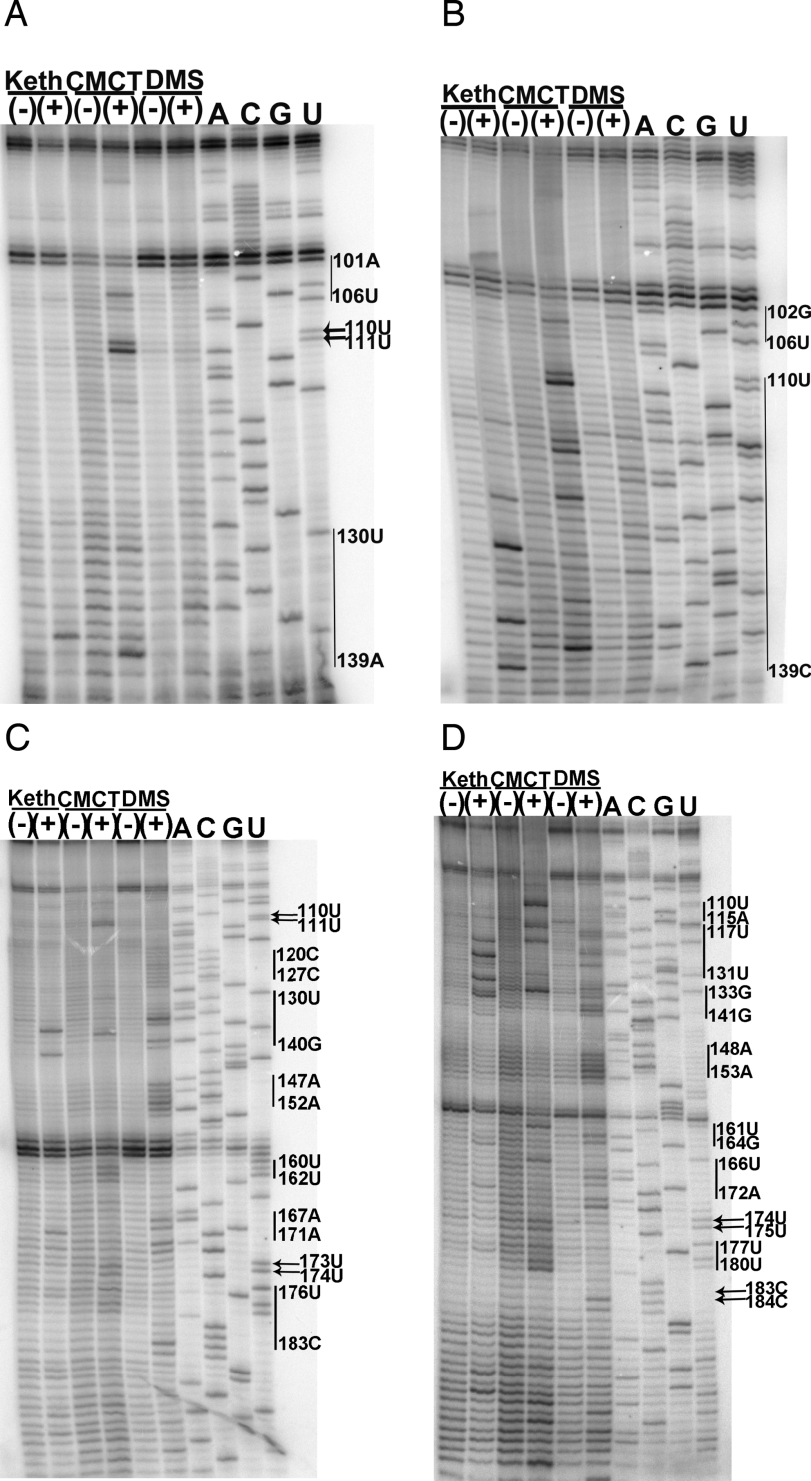
Chemical probing results for CV-B3/28 and CV-B3/GA SLII. For all sequencing gels, arrows indicate modified positions and vertical lines between two nucleotide positions indicate that all positions between and including the positions shown are modified. Sequencing tracks indicating the nucleotide positions are labelled A, C, G and U. Tracks with chemically modified RNA are labelled (+) and tracks with unmodified RNA are labelled (−). (**A**) 12% sequencing gel shows CV-B3/28 SLII region–positions 80–140. (**B**) 12% sequencing gel shows CV-B3/GA SLII region–positions 80–140. (**C**) 12% sequencing gel shows CV-B3/28 SLII region–positions 110–190. (**D**) Chemical probing of CV-B3/GA SLII region–positions 110–200.

**Figure 2.**
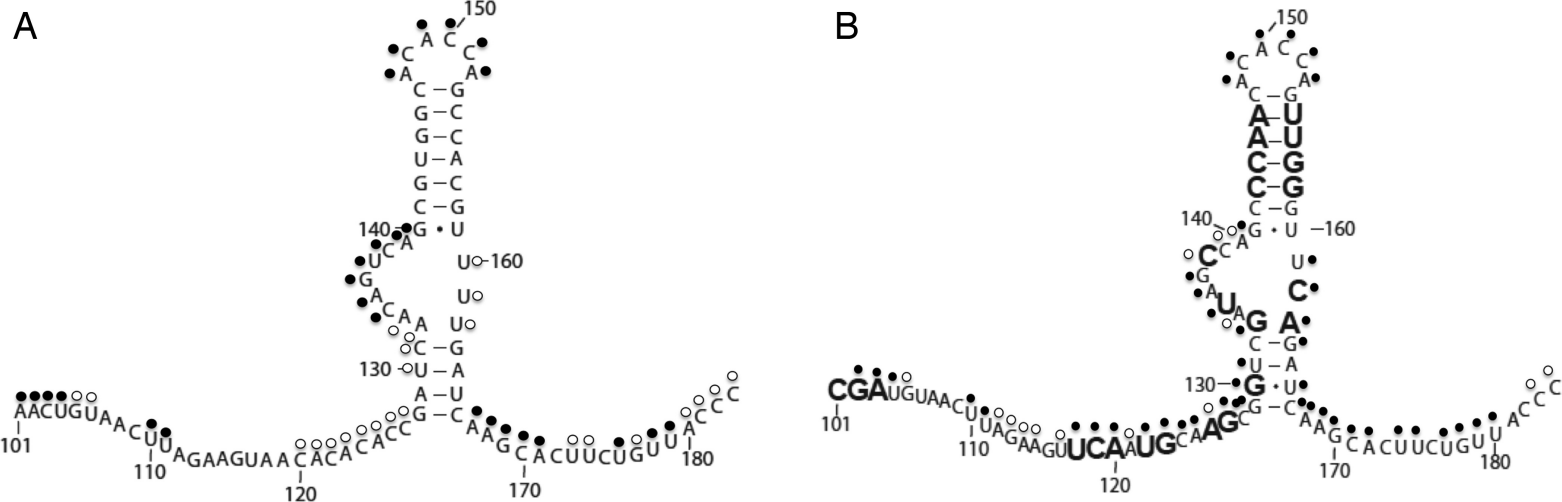
Predicted secondary structure model of CV-B3/28 and CV-B3/GA SLII. (**A**) Predicted secondary structure model of CV-B3/28 SLII. (**B**) Predicted secondary structure of CV-B3/GA SLII. Enlarged nucleotide positions are substituted or inserted in CV-B3/GA.Modified positions are indicated with a circle. Closed circles indicate heavily modified positions and open circles indicate moderately modified positions.

The structure of the SLII region was analysed with chemical probing analysis (Figure 1). A summary of the modification results at each position is displayed on models of SLII in Figure 2. The C-rich region ranging from positions 90–100 is shown at the top of Figure 1A and B. A strong stop at positions 99–100 partially obscures the results for the 90–100 region in Figure 1A and B. Despite the strong stop, a lack of reactivity is observed in the DMS lane for both CV-B3/28 and CV-B3/GA between positions 90 - 100, indicating that this C-rich region is not accessible to solvent. The A-U rich region spanning positions 100—110 is partially protected in both CV-B3/28 and CV-B3/GA (Figure 1A and B). Positions 102G, 103A, 104U, 105G and 106U are modified in CV-B3/GA. Likewise, for CV-B3/28, positions 101A, 102A, 103C, 104U, 105G and 106U are accessible.

Heavy modifications were observed in both CV-B3/GA and CV-B3/28 at positions 110U and 111U (Figure 1A and B). Just downstream of positions 110U and 111U is the region with the highest density of sequence changes between the CV-B3/GA and CV-B3/28 5′UTRs. Between positions 118 and 147, 15 of the 30 nucleotides are substituted in CV-B3/GA (Figure 2A and B). A major structural difference between CV-B3/GA and CV-B3/28 is observed in this region, as indicated by the drastic difference in chemical modification patterns in the middle of the gel shown in Figure 1A and B as well as on the top of the gel shown in Figure 1C and D. In CV-B3/28, only positions 120–127 are modified and the modifications are weak (Figure 1A and C). In sharp contrast, every position between 110 and 141, 19 of the 24 positions between 161 and 184 and positions predicted to pair such as 129G, 130G, 131U, 164G, 166U and 167C are heavily modified in CV-B3/GA (Figure 1B and D).

Chemical probing results for the entirety of CV-B3/28 and CV-B3/GA SLII are shown in Figure 1C and D. These results were generated using a primer that is downstream of SLII and distinct from the primer used to generate the results shown in Figure 1A and B. The drastic structural difference between CV-B3/GA and CV-B3/28 in the 110–130 region is once again clearly shown in the top portion of Figure 1C and D. Downstream of this region, CV-B3/28 and CV-B3/GA have conserved structure from positions 134–160. This structural conservation occurs despite a continued high frequency of substitutions. Positions 141–146 are predicted to base pair with positions 154–159 to form the upper helical stem of SLII (according to CV-B3/GA sequence position numbering) (Figure 2). These positions are unmodified, matching the model's predictions for both CV-B3/28 and CV-B3/GA (Figure 1C and D). Interestingly, 8 of the 12 positions forming the SLII upper stem are substituted in CV-B3/GA. These substitutions occur in compensatory pairs that are predicted to maintain Watson–Crick base pairing. Our probing results confirm that these compensatory changes do indeed allow conserved helical structure. The sequence of the apical loop region (147A, 148C, 149A, 150C, 151C, 152A, according to CV-B3/28 sequence position numbering) is identical in CV-B3/28 and CV-B3/GA and all six nucleotides are similarly accessible in both 5′UTRs (Figure 1C and D).

The chemical probing results for the 3′ side of SLII are also shown in Figure 1C and D. The upper helical stem, including positions 153–158, is protected in both molecules. Positions 159–161 are exposed in both CV-B3/GA and CV-B3/28, matching the structural models (Figure 2A and B) that show these positions forming the 3′ side of the asymmetric internal loop. For CV-B3/28, positions 163–166 are protected (Figure 1C). The inaccessibility of CV-B3/28 positions 163–166 further supports the model for a CV-B3/28 domain II lower stem (Figure 2A). In contrast, for CV-B3/GA, 165A is the only position in the lower stem region (163–167, according to CV-B3/GA sequence) that is not modified (Figure 1D). This difference in modification agrees with the results observed for the 5′ side of the SLII lower stem (Figure 1A and B) where CV-B3/GA is exposed and CV-B3/28 is protected. Together these results suggest a lower stem (128–132 and 162–166) forms in CV-B3/28 SLII but fails to form in CV-B3/GA SLII. The frequent and heavy modification in CV-B3/GA suggests that the only structure present in CV-B3/GA SLII is the upper stem loop (positions 142–160, according to CV-B3/GA sequence). On the other hand, our modification results show that there is extensive structure in both the upper and lower regions of SLII in CV-B3/28.

### Comparative sequence analysis of SLII

**Figure 3.**
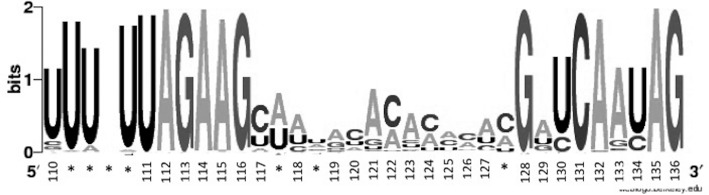
Sequence logo showing conservation between positions 110–136 in 170 aligned 5′UTR sequences. The numbers on the x-axis correspond to nucleotide positions found in the CV-B3/28 5′UTR. Positions where the alignment shows insertions in the CV-B3/28 sequence are labelled with *. The level of conservation at each position is measured in bits on the y-axis where two bits indicate the highest level of conservation at each position.

The lack of conservation and the structural differences between CV-B3/28 and CV-B3/GA in the SLII region raised questions about this region's sequence conservation among other enteroviruses. We compared the sequence of this region in 170 diverse enteroviruses including coxsackieviruses, polioviruses and numbered enteroviruses. Representatives of *Enterovirus A, Enterovirus B* and *Enterovirus C* were included in the analysis. Only full-length genomes were included in the alignment. The genomes were aligned and the region spanning 117–127 was not conserved. This is illustrated in a sequence logo (Figure 3), where the short letters correspond to poorly conserved regions and tall letters correspond to highly conserved regions. The CV-B3/28 nucleotide position numbering is shown in the sequence logo x-axis. Interestingly, the largest single stretch of poor SLII sequence conservation corresponds to the region with the most sequence differences between CV-B3/28 and CV-B3/GA and also corresponds with the region where we detect major structural differences in the 5′UTRs.

### Structural comparison of SLII chimeras

**Figure 4.**
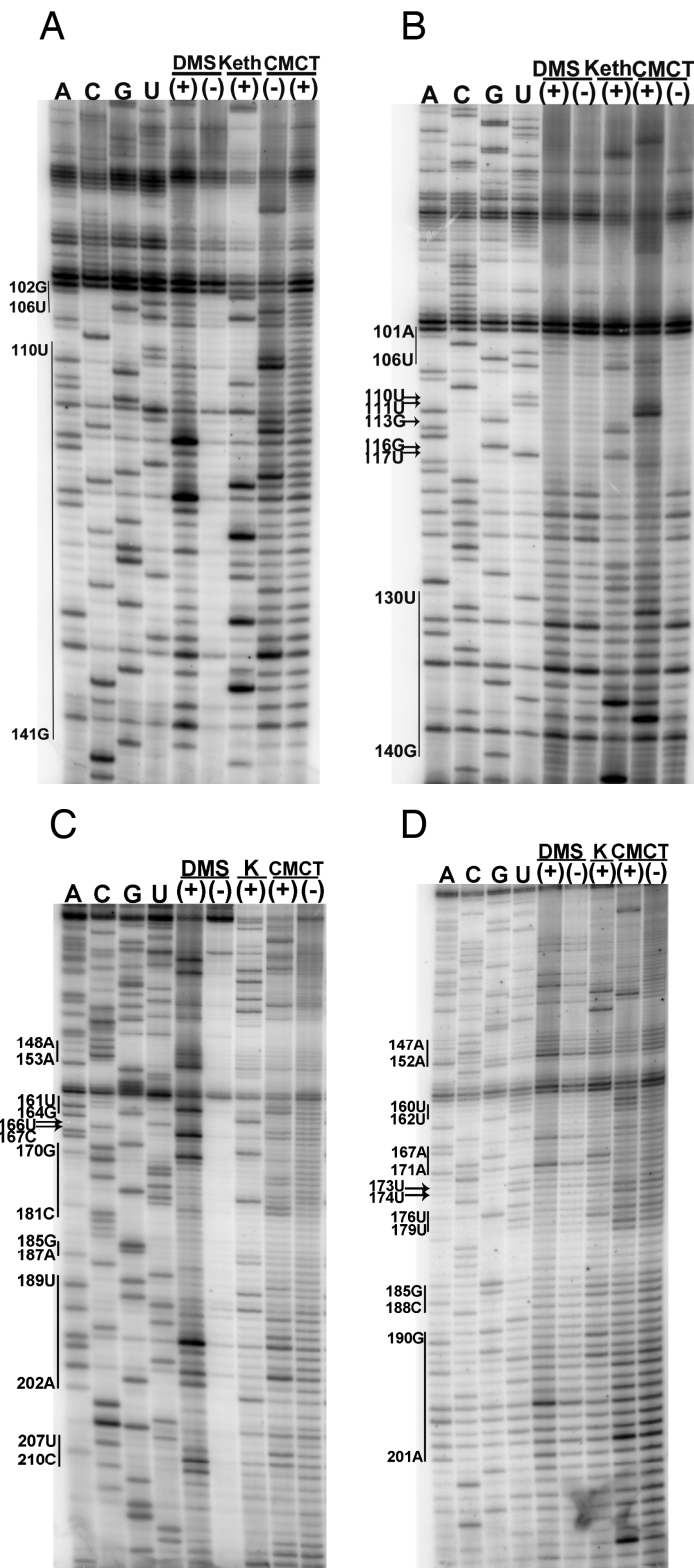
Chemical probing results for 28 SLII GA and GA SLII 28 chimeras. Positions 104–180 were exchanged between CV-B3/28 and CV-B3/GA. For all sequencing gels, arrows indicate modified positions and vertical lines between two nucleotide positions indicate that all positions between and including the positions shown are modified. Sequencing tracks indicating the nucleotide positions are labelled A, C, G and U. Tracks with chemically modified RNA are labelled (+) and tracks with unmodified RNA are labelled (−). (**A**) 12% sequencing gel showing 28 SLII GA (CV-B3/28 5′UTR with CV-B3/GA SLII). (**B**) 12% sequencing gel showing GA SLII 28 (CV-B3/GA 5′UTR with CV-B3/28 SLII). (**C**) 12% sequencing gel showing 28 SLII GA. (**D**) 12% sequencing gel showing GA SLII 28.

To further explore the 5′UTR RNA structure of virulent and avirulent isolates, SLII (104–184) was exchanged between CV-B3/28 and CV-B3/GA to generate the chimeric constructs 28 SLII GA (CV-B3/28 5′UTR with CV-B3/GA SLII) and GA SLII 28 (CV-B3/GA 5′UTR with CV-B3/28 SLII). As found in the parent molecules, 28 SLII GA was heavily modified in the SLII region (Figure 4A and C) and GA SLII 28 was protected (Figure 4B and D). Thus, probing results for 28 SLII GA matched that of the non-chimeric CV-B3/GA SLII and probing results for GA SLII 28 matched that of the non-chimeric CV-B3/28. The other five 5′UTR structural domains in 28 SLII GA and GA SLII 28 were also analysed with chemical probing analysis and no changes occurred in the structural domains outside of SLII in either chimera (data not shown).

### Structural comparison of domains I, III, IV, V and VI

**Figure 5.**
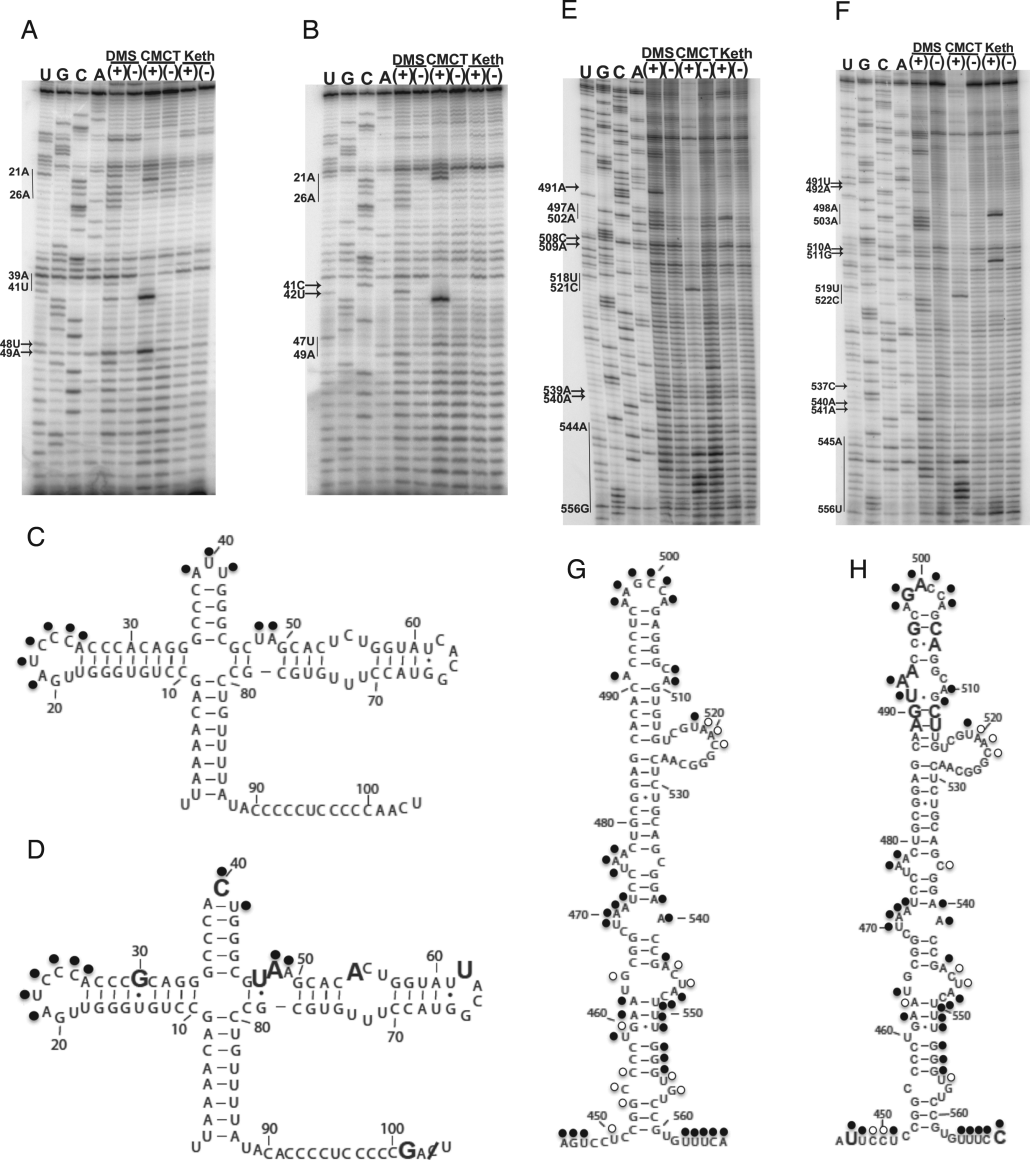
Chemical probing results for CV-B3/28 and CV-B3/GA domains I and V. For all sequencing gels, arrows indicate modified positions and vertical lines between two nucleotide positions indicate that all positions between and including the positions shown are modified. Sequencing tracks indicating the nucleotide positions are labelled A, C, G and U. Tracks with chemically modified RNA are labelled (+) and tracks with unmodified RNA are labelled (−). (**A**) 12% sequencing gel showing CV-B3/28 domain I. (**B**) 12% sequencing gel showing CV-B3/GA domain I. (**C**) Predicted secondary structure model of CV-B3/28 domain I. Modified positions are indicated with a circle. Closed circles indicate heavily modified positions and open circles indicate moderately modified positions. (**D**) Predicted secondary structure model of CV-B3/GA. Modified positions are indicated with a circle. Closed circles indicate heavily modified positions and open circles indicate moderately modified positions. Enlarged nucleotide positions are substituted or inserted in CV-B3/GA. The position with the slash is deleted in CV-B3/GA. (**E**) 12% sequencing gel showing CV-B3/28 domain V. (**F**) 12% sequencing gel showing CV-B3/GA domain V. (**G**) Predicted secondary structure model of CV-B3/28 domain V. Modified positions are indicated with a circle. Closed circles indicate heavily modified positions and open circles indicate moderately modified positions. (**H**) Predicted secondary structure model of CV-B3/GA domain V. Modified positions are indicated with a circle. Closed circles indicate heavily modified positions and open circles indicate moderately modified positions. Enlarged nucleotide positions are substituted in CV-B3/GA.

The structure of domains I, II, III, IV, V and VI for both CV-B3/28 and CV-B3/GA were determined with chemical probing analysis. The chemical probing results for domain I (Figure 5A–D) and domain V (Figure 5E–H) are shown in Figure 5 as representatives of the overall comparison. No major structure differences between CV-B3/GA and CV-B3/28 were observed in domains I, III, IV, V or VI. Minor alterations were observed in the structural domains outside of SLII, however these changes were highly localized when compared to the clear differences observed in SLII.

## DISCUSSION

The region connecting the structural domains I and II together with domain II has been collectively referred to as the SLII region. SLII is a virulence determinant in several enteroviruses including CV-B3, PV, CVB1 and enterovirus 71 (EV71) (33–40). The importance of SLII to PV neurovirulence was demonstrated in a series of studies showing a drastically reduced PV neurovirulence in mutant strains with substitutions in positions 128–134 (28,38–40). Chimeric constructs with SLII exchanged between virulent and avirulent CVB1 strains demonstrated that SLII is also a CVB1 virulence determinant (33). More recently, SLII has been proven critical to EV71 with similar experiments that observed SLII exchanged chimeric constructs and found virulence is determined by SLII (36).

Avirulent phenotypes have been observed in enterovirus mutants with substitutions and deletions in several different regions of the 5′UTR (11,49,46). The attenuation phenotype is widely believed to be the result of disruption in 5′UTR secondary and tertiary RNA structures that are required for recruiting cellular and viral protein factors (52–54). The importance of the 5′UTR secondary and tertiary structural integrity is widely acknowledged, however there are only a few studies with experimental data definitively showing how mutations in the 5′UTR change critical structures (55,56).

In this study, the 5′UTR structure of a virulent CV-B3/28 strain and an avirulent CV-B3/GA strain were compared using chemical probing analysis (51). CV-B3/GA was an ideal strain for comparison given its natural attenuation and previous characterization by Lee *et al.* (49). In that characterization of CV-B3/GA, energy minimization of RNA folding (57) was used to predict that the CV-B3/GA SLII region secondary structure was altered in comparison to cardiovirulent CV-B3 strains. The experimental results presented here confirm a SLII structural difference between CV-B3/28 and CV-B3/GA, however they do not return the same structural alteration predicted by energy minimization. As has been found in other studies of RNA folding, experimental approaches are required to elucidate the biologically relevant structure (58).

**Figure 6.**
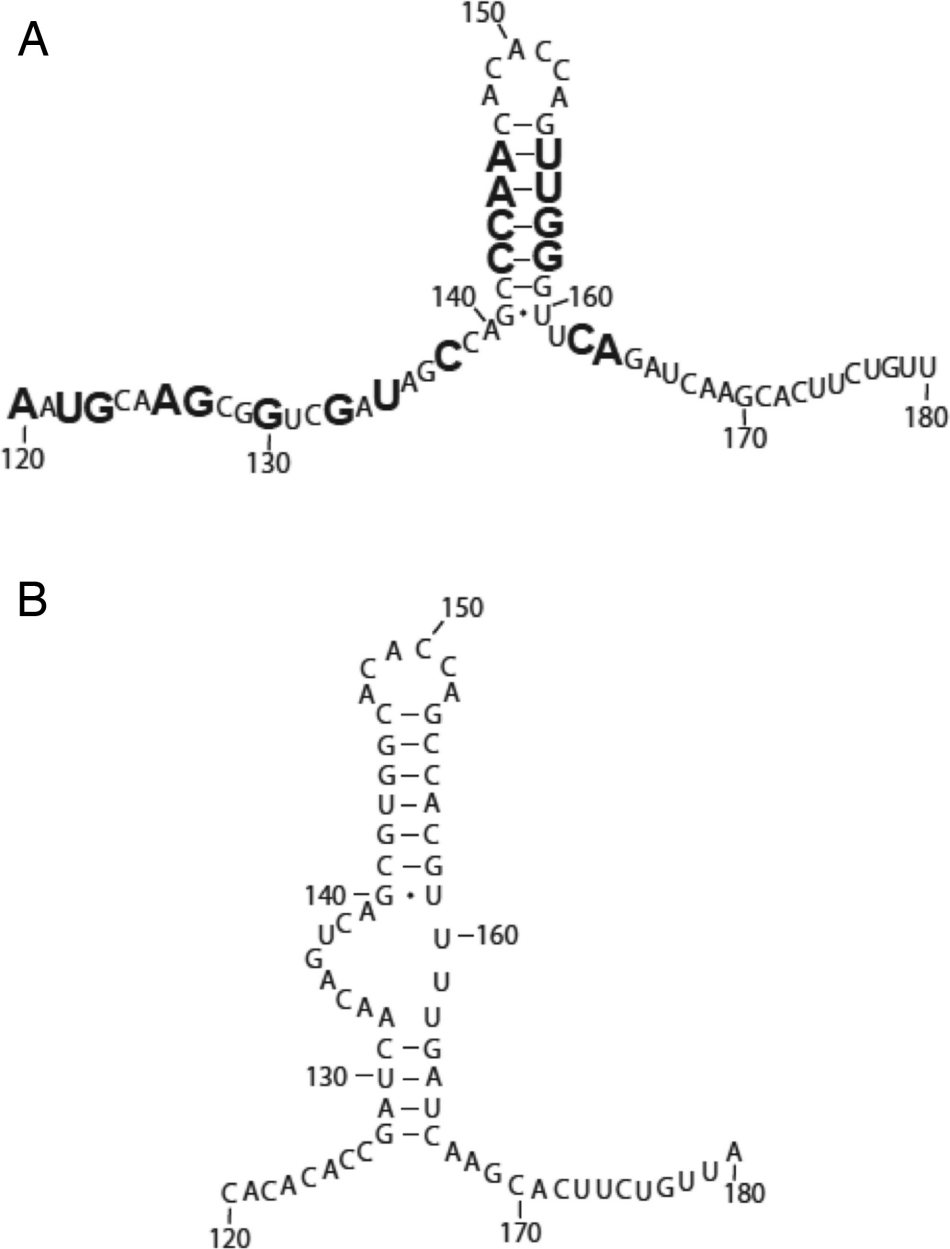
Models for structurally distinct domain II folding in CV-B3/GA and CV-B3/28. (**A**) CV-B3/GA. (**B**) CV-B3/28. The enlarged nucleotide positions are substituted or inserted in CV-B3/GA.

Comparison of the full length naturally folded 5′UTRs of CV-B3/GA and CV-B3/28 revealed that a drastic structural alteration occurs in the SLII region. CV-B3/28 SLII includes pairing between positions 128–132 with 162–166 forming a lower stem that does not form in CV-B3/GA (Figure 6). These results may serve as a starting place to understanding the SLII structure required for enterovirus cardiovirulence.

This study also sheds some light on the folding behaviour of SLII and structural interdependence of the 5′UTR as a whole. The loosely defined SLII region including positions 104–184 was exchanged between CV-B3/GA and CV-B3/28 and the chemical probing analysis of the full length 5′UTR was repeated with the chimeric constructs. Collectively, the chemical probing analysis of the chimeras shows that the secondary structure of SLII follows the parent molecule and does not depend on the 5′UTR structures outside of the SLII region. Similarly the structure of the 5′UTR domains outside of SLII are not influenced by the SLII exchange. Overall these results show that the 5′UTR structural domains fold independently. These results also provide a structure-based explanation to previous reports showing that cardiovirulence of SLII chimeras follows SLII of the parent strain. These and past results suggest that SLII is both structurally and functionally independent as a cardiovirulence determinant.

The natural attenuation of CV-B3/GA and the results of this study raise interesting questions about the overall role of SLII in CV-B3 virulence. In addition to further supporting SLII as a cardiovirulence determinant, the results of this study also suggest that SLII mediates virulence without being critical for viral viability. First, the only structural differences we detected between CV-B3/28 and CV-B3/GA occur in SLII with the remainder of the 5′UTR structure conserved. We acknowledge the limits of chemical probing analysis and that this technique primarily distinguishes between Watson–Crick base paired positions versus non-Watson–Crick base paired positions, however our results clearly indicate structural conservation among the other five domains. The maintenance of the structural integrity in the domains outside SLII may therefore be required for viability. Second, we included comparative analysis of 170 enteroviruses including the region where the major structural alteration is observed. There is no conservation in the region where the structural alteration occurs, once again suggesting that the primary structure and the higher order structures dependent on these primary structures may not be important to overall enterovirus viability and only important to cardiovirulence. Finally, the probing analysis of the chimeras showed that SLII folds independently. The structural independence of SLII would allow alterations in this region to occur without altering or undoing structural elements in other regions of the 5′UTR that are essential for viability.

## Supplementary Material

SUPPLEMENTARY DATA
