# Supplementary material for: Major alteration in coxsackievirus B3 genomic RNA structure distinguishes a virulent strain from an avirulent strain
Source: Nucleic Acids Res. 2014 Jul 29;42(15):10112–21. doi: 10.1093/nar/gku706 (PMC4150801; doi:10.1093/nar/gku706)
Supplement: SUPPLEMENTARY DATA [file supp_42_15_10112__index.html]

The bacterial antitoxin HipB establishes a ternary complex with operator DNA and phosphorylated toxin HipA to regulate bacterial persistence — The bacterial antitoxin HipB establishes a ternary complex with operator DNA and phosphorylated toxin HipA to regulate bacterial persistence — The bacterial antitoxin HipB establishes a ternary complex with operator DNA and phosphorylated toxin HipA to regulate bacterial persistence — SUPPLEMENTARY DATA 

# Major alteration in coxsackievirus B3 genomic RNA structure distinguishes a virulent strain from an avirulent strain

## SUPPLEMENTARY DATA

**Files in this Data Supplement:**

- SUPPLEMENTARY DATA
