# Supplementary material for: Major alteration in coxsackievirus B3 genomic RNA structure distinguishes a virulent strain from an avirulent strain
Source: Nucleic Acids Res. 2014 Jul 29;42(15):10112–21. doi: 10.1093/nar/gku706 (PMC4150801; doi:10.1093/nar/gku706)
Supplement: SUPPLEMENTARY DATA [file supp_gku665_nar-00886-h-2014-File006.pdf]

The bacterial antitoxin HipB establishes a ternary complex with operator DNA and phosphorylated toxin HipA to regulate bacterial persistence

Yurong Wen[1,2], Ester Behiels[1,3], Jan Felix[2], Jonathan Elegheert[2,3], Bjorn Vergauwen[2], Bart Devreese[1*], Savvas N Savvides[2*]

[1] Unit for Biological Mass Spectrometry and Proteomics, Laboratory for Protein Biochemistry and Biomolecular Engineering (L-ProBE), Ghent University, K.L. Ledeganckstraat 35, 9000 Ghent, Belgium.
[2] Unit for Structural Biology, Laboratory for Protein Biochemistry and Biomolecular Engineering (L-ProBE), Ghent University, K.L. Ledeganckstraat 35, 9000 Ghent, Belgium.
[3] Present Address: Division of Structural Biology, Wellcome Trust Centre for Human Genetics, University of Oxford, Roosevelt drive, OX3 7BN, Oxford, UK.
To whom correspondence should be addressed: Savvas N Savvides E-mail: Savvas.Savvides@Ugent.be ; Tel: +32 472 92 85 19, and Bart Devreese E-mail: Bart.Devreese@Ugent.be ; Tel:+32 9 264 52 73.

SUPPLEMENTARY MATERIALS

Figure S1. Sequence alignment of *S. oneidensis* MR-1 and *E. coli* K12 HipA. HipA (SO0706) and HipA (*E. coli*) share a 28% sequence identity. The sequences underlined in orange indicate the 5 loops involved in AMPPNP and Mg^{2+} binding.

```
HipA_SO0706   1 MSTAKTLTLEMHLGDLMIGELSFDATADTFAVHYTKDWQQSGFP--LSPTIPLD-GTGTSNQIS 61
HipA_E.coli   1 -----MPKLVTWMNNQRVGELTKLANG-AHTFKYAPEWLASRYARQLSLSLPLQRGNITSDAVF 58

HipA_SO0706  62 MFLVNLLPEN-KGLDYLIESLGVSKGNTFALIRAIGLDTAGAIAFVPKG-----ALLPETQLRP 119
HipA_E.coli  59 NFFDNLLPDSPIVRDRIVKRYHAKSRQPFDLLSEIGRDSVGAVTLIPEDETVTHPIMAWEKLTE 122

HipA_SO0706 120 IKAEEVIQRIEDPTMWPMEIWDGKPRLSVAGVQPKLNLFYNGKEFAFAEGTLSSTHIVKFEK-- 181
HipA_E.coli 123 ARLEEVLTAYKADIPLGMIREENDFRISVAGAQEKTALLRIGNDWCIPKGITPTTHIIKLPIGE 186
                                            Loop1

HipA_SO0706 182 ---------YHHLVINEFITMRLAKVLGMNVANVDIVHFGRYKALCVERFDRRNIPGEQRVLRR 236
HipA_E.coli 187 IRQPNATLDLSQSVDNEYYCLLLAKELWVNVPDAEIIKAGNVRALAVERFDRRWNAERTVLLRL 250
                                                              Loop2

HipA_SO0706 237 HIVDSCQALGFSVSKKYERNFGTGRDVKDIREGVSFNRLFSLAAKCRNPVAAKQDMLQWALFNL 300
HipA_E.coli 251 PQEDMCQTFGLPSSVKYESDGGP-----------RIARIMAFLMGSSEALKDRYDFMKFQVFQW 303
                              Loop3

HipA_SO0706 301 LTGNADAHGKNYSFFMTPSG-MEPTPWYDLVSVDMYED------FEQQLAMAIDDEFDP----N 353
HipA_E.coli 304 LIGATDGHAKNFSVFIQAGGSYRLTPFYDIISAFPVLGGTGIHISDLKLAMGLNASKGKKTAID 367
                     Loop4                Loop5

HipA_SO0706 354 SIYAYQLAAFMDGLGLPRNLLISNLTRIARRIPQAIAEVILMLPP-LDEDEASFVAHYKTQLLA 416
HipA_E.coli 368 KIYPRHFLATAKVLRFPEVQMHEILSDFARMIPAALDNVKTSLPTDFPENVVTAVESNVLRLHG 431

HipA_SO0706 417 RCERYLGFVDEVRDVEV                                                433
HipA_E.coli 432 RLSREYGSK--------                                                440
```

Figure S2. a. Comparison of *hipAB* operator sequences in *S. oneidensis* MR-1 and *E. coli*. The 4 operator sequences are underlined and conserved sequences are indicated in bold face. The start codon of *hipB* is highlighted in red. The start codons for both *hipA* and *hipB* in *S.oneidensis* MR-1 are predicted to be TTG. The *S. oneidensis* MR-1 operator GTGTA(N6)TACAC (N34)GTGTA(N6)TACAC(N34)GTGTA(N6)TACAC(N34)GTGTA(N6)TACAC and the *E. coli* operators TATCC(N8)GGATA(N10)TATCC(N8)GGATA(N21)TATCC(N8)GGATA(N10) TATCC(N8)GGATA. **b.** Operon organization of $HipAB_{so}$ from *S. oneidensis* MR-1. (A) *S. oneidensis* total cDNA template and primers for *hipB*. (B) *S. oneidensis* total cDNA template and primers for *hipA*. (C) *S. oneidensis* total cDNA template and primers for *hipAB*. (Neg. Ctrl. =negative control, using no-RT RNA template to asses DNA contamination in RNA preparations; Pos.Ctrl. = positive control, using *S. oneidensis* genomic DNA template). **c.** Overexpression of $HipA_{so}$ results in temporary inhibition of growth in *S. oneidensis* MR-1. Left panel: overexpression of $HipA_{so}$, $HipB_{so}$ and $HipAB_{so}$ in the wild type *S. oneidensis* MR-1; right panel: overexpression of $HipB_{so}$, $HipAB_{so}$ in the $\Delta HipAB_{so}$ mutant *S.oneidensis* MR-1. At least three independent growth curves were evaluated for each experiment. Error bars indicate the standard error of mean.

a

***S. oneidensis* MR-1**

CGACTTGGGTAAGTATTAG**GTGTA**CTTATC**TACAC**TTTTTGGGTTTTAAGCACTGAA
ATCGCTATGAAG**GTGTA**ACATTG**TACAC**TTTTGGTTGGCTTGTCATTATCAACAATC
AAAAA**GTGTA**CTTATA**TACAC**TTTTTGATTGAATGGGACTGATATTAAGGCTAAG**G
TGTA**TGAAGA**TACAC**TTTTGGAGACGATAATGGCA

***E. coli* K12**

TTAAATCCTCCTTTT**TATCC**GCGATCGC**GGATA**TCGCAGCGTT**TATCC**CGTAGAGC**G
GATA**AGATGTGTTTCCAGATTGACT**TATCC**TCACTAAA**GGATA**AAACTTATAA**TAT
CC**CCTTAAGC**GGATA**AACTTGCTGTGGACGTATGACATGATGAGCTT

b

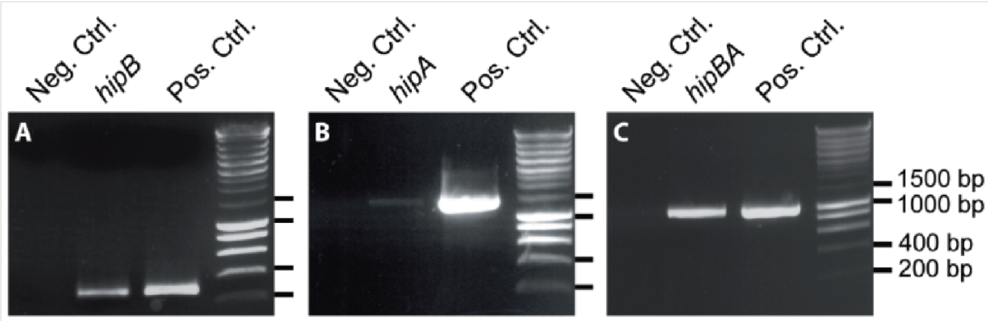

c

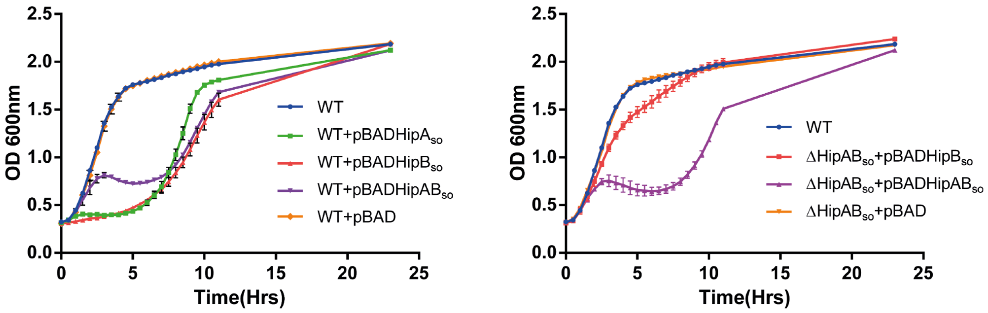

Figure S3. a. ITC experiments of HipB with DNA containing 2 operators (76bp) and N termini (Nt) truncated $HipB_{so}$ with $HipA_{so}$ **b.** One Protein:DNA interaction sites in the ternary $HipA_{so}$-$HipB_{so}$:DNA complex plotted by NUCPLOT. **c.** Interactions of the $HipB_{so}$ C-terminal G95-W96-Y97 motif with $HipA_{so}$. Red circles indicate $HipA_{so}$ hydrophobic interactions.

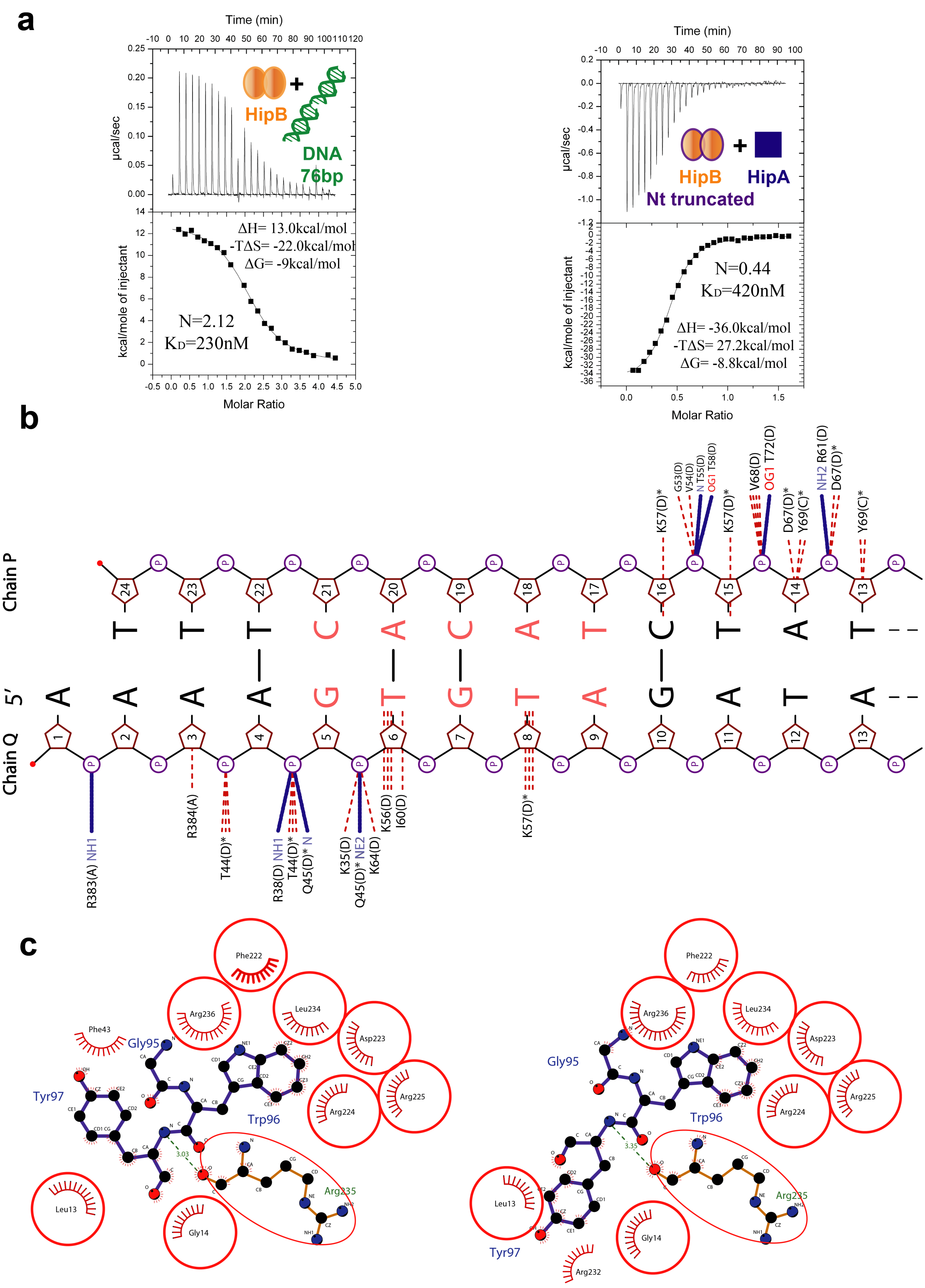

Figure S4. a. Details of the interaction between $HipA_{so}$, AMPPNP, Mg^{2+} and water molecules. The blue balls represent the water molecules involved in the interaction. **b.** Binding of AMPPNP to $HipA_{so}$. 2Fo-Fc density is shown at 2σ. Surface view of the $HipA_{so}$-AMPPNP-Mg^{2+} structure; the pocket for ATP binding is outlined in orange. **c.** Structural alignment of $HipA_{so}$ (AMPPNP-Mg^{2+} bound form) and phosphorylated $HipA_{so}$ extracted from the $HipAB_{so}$:DNA complex reveals ejection of the pLoop. The pLoop is shown in red (pHipA), magenta (AMPPNP-Mg^{2+} bound HipA) with the phosphorylation sites Ser147 shown as colored spheres. In the $HipB_{so}$-bound form, the $HipA_{so}$ C-terminal helix bundle, essential for DNA binding, is shifted by 2Å.

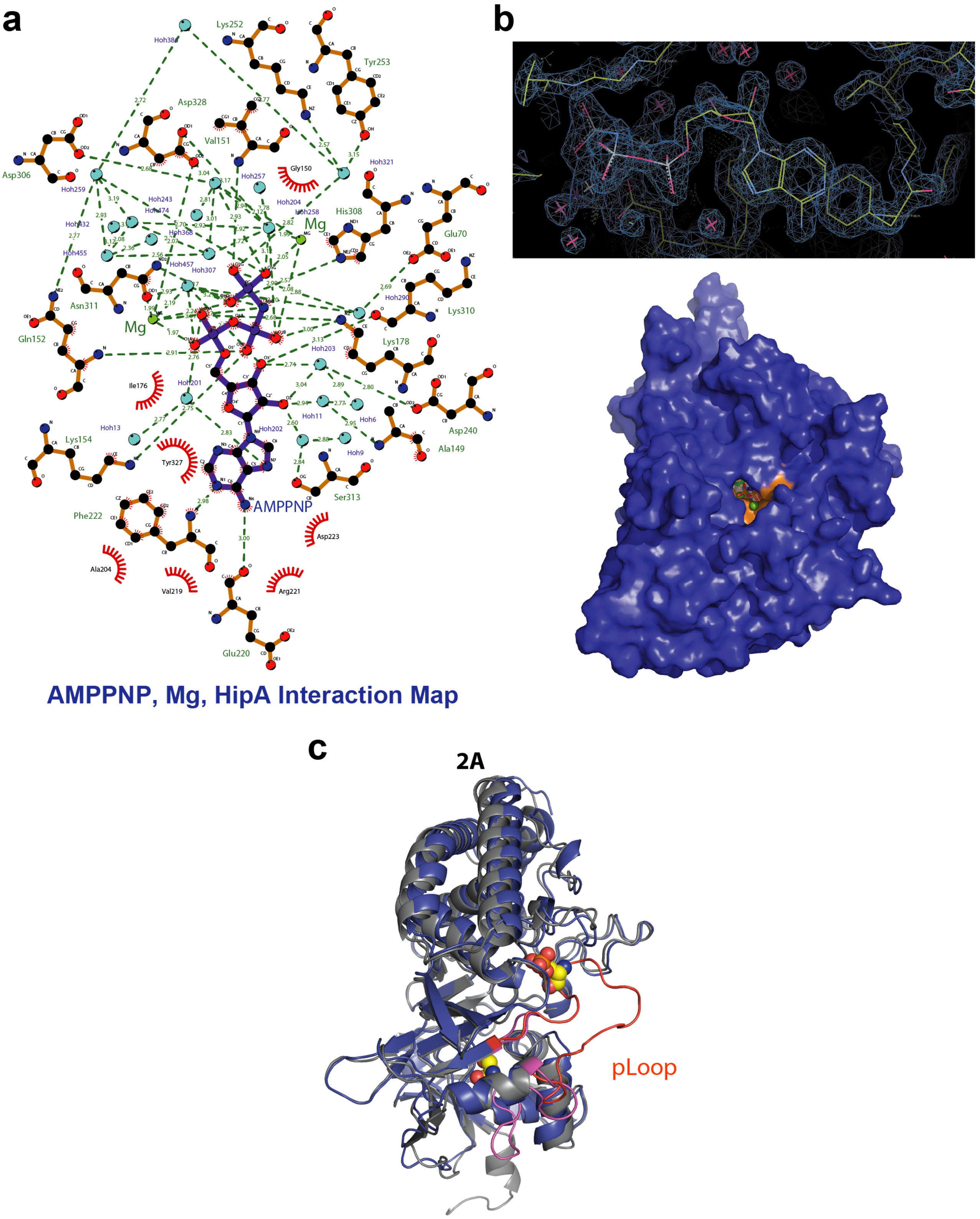

Figure S5. a. *In vitro* radioactive kinase assay. No phosphate transfer between *S. oneidensis* MR-1 $HipA_{so}$ and $EF\text{-}Tu_{so}$ could be detected. A 50 kDa band can be seen corresponding to autophosphorylation of $HipA_{so}$, while this band is absent for the active site mutant $HipA_{so}D306Q$. Left: Autoradiograph. Right: SDS-PAGE gel. $HipA_{so}$ was used as a positive control and $HipA_{so}D306Q$ was used as a negative control in this experiment. Lane 1 $HipA_{so}D306Q$, 2 $HipA_{so}$, 3 $HipA_{so}$+$EF\text{-}Tu_{so}$-His, 4 $HipA_{so}$+$EF\text{-}Tu_{so}$-GST, 5 $HipA_{so}$+$EF\text{-}Tu_{so}$-His+GDP, 6 $HipA_{so}$+$EF\text{-}Tu_{so}$-GST+GDP, 7 $HipA_{so}D306Q$+$EF\text{-}Tu_{so}$-His+GDP 8 $HipA_{so}D306Q$+$EF\text{-}Tu_{so}$-GST+GDP. 9 $EF\text{-}Tu_{so}$-His, 10 $HipA_{so}$, 11 $EF\text{-}Tu_{so}$-GST. **b.** ITC experiment reveals no apparent interaction between $HipA_{so}$ and $EF\text{-}Tu_{so}$.

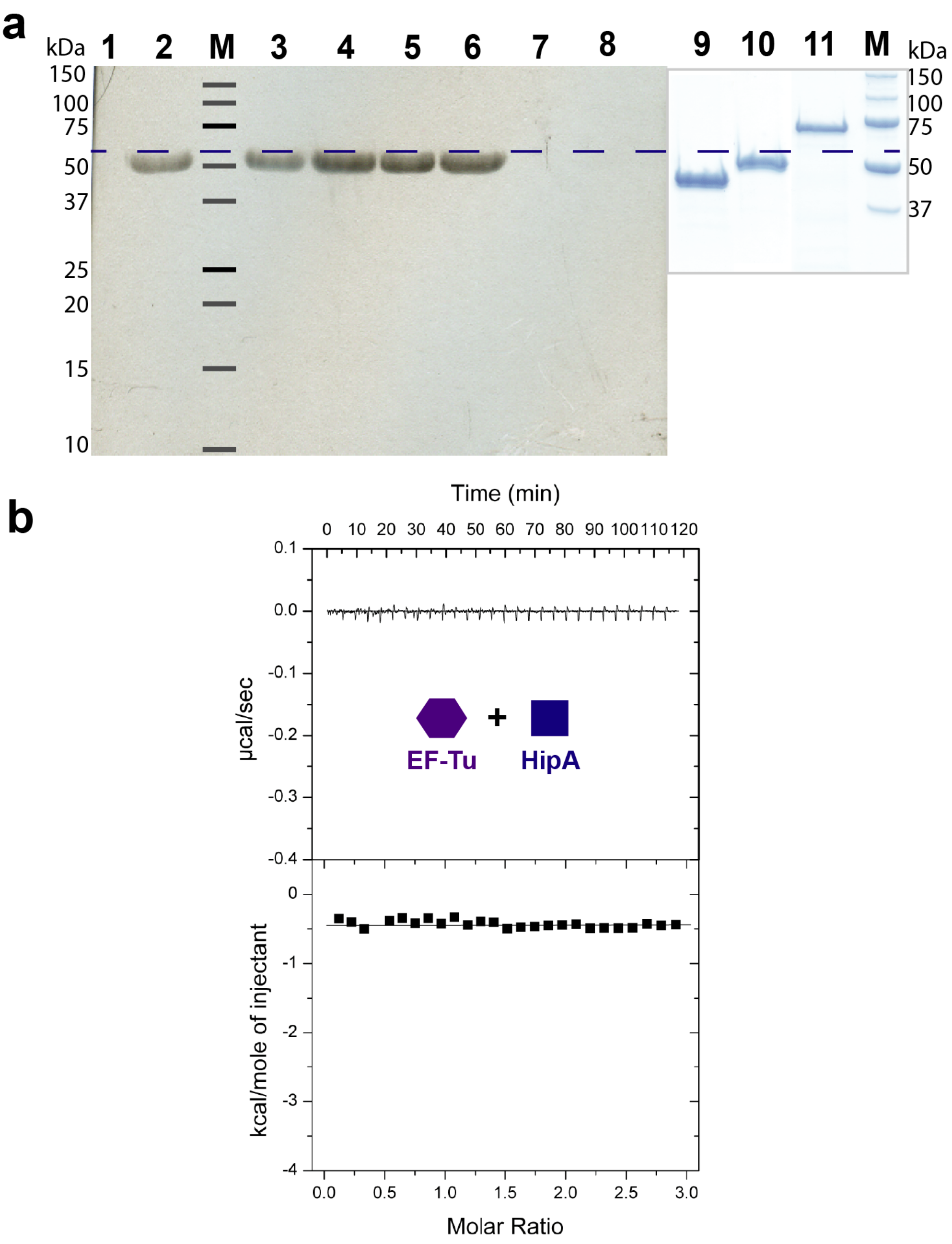

Figure S6. Sequence alignment of SO0706 ($HipA_{so}$), SO3170 and SO0063. The potential Ser phosphorylation site is marked with a green star and the residues involved in ATP and Mg^{2+} binding are marked with red stars. The dark and lighter blue colors indicate the level of conservation.

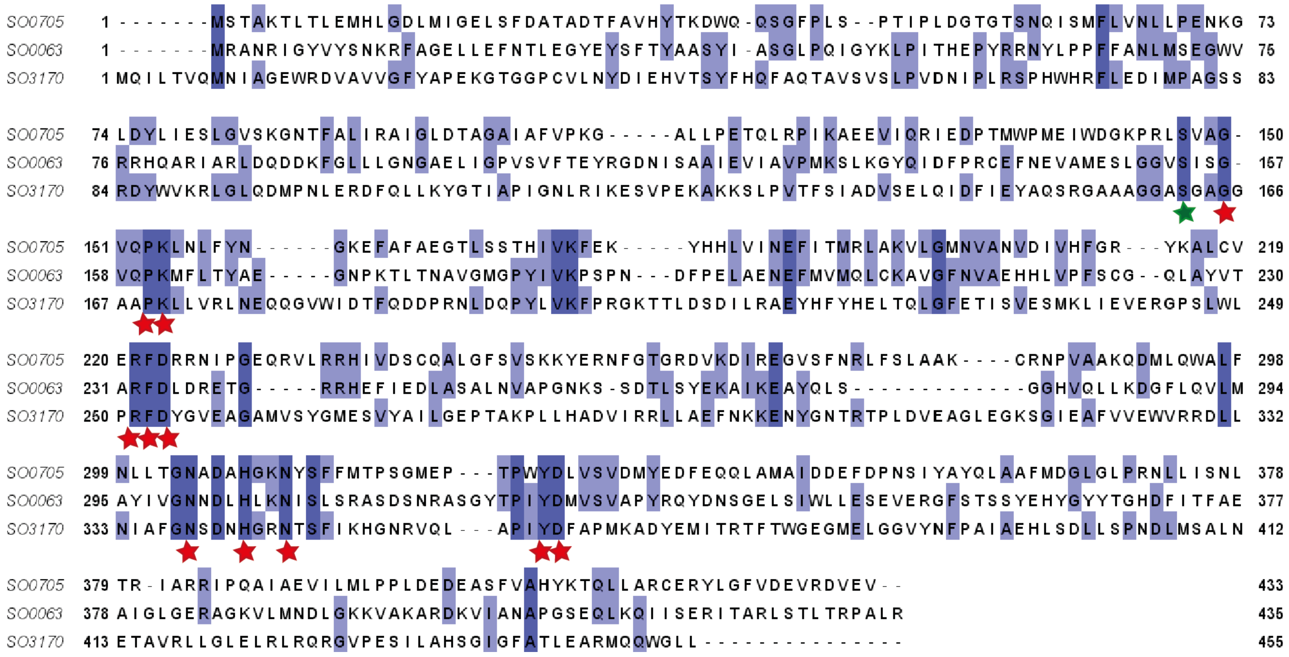

Table S1. Primers used in this study.

Name	Sequence(5'-3')
**MR01**	CATATGAGTACAGCTAAAACGCTTACG
**MR02**	GGATCCCTACACTTCCACATCCCTGAC
**MR03**	CATATGAATGGGACTGATATTAAGG
**MR04**	GGATCCTTAATACCAGCCGTTAGTTTC
**MR05**	ACCGGCAATGCCCAAGCACACGGTAAAAACTACTC
**MR06**	GAGTAGTTTTTAC CGTGTGCTTGGGCATTGCCGGT
**MR07**	CATATGGCAAAAGCTAAATTTGAACG
**MR08**	CTCGAGGCAGCAATGATCTTAGCTACTACAC
**MR09**	TGGCTGCAGTGGGCGGCATGTGAGTGTATCCTAGCTGGTTTA
**MR10**	TGTGAATTCATACACCTTAGCCTTAATATCAGTCCCATT
**MR11**	GCTGAATTCAGCGACACCGAAACTAACGGCTGGTATTAA
**MR12**	CTTGGATCCCCGCAATTGTGTTTCAGGCAGTAAAGCCCC
**MR13**	TCAGGATCCAAAAAGTGTACTTATATACACTTTTTGA
**MR14**	CGTGAATTCAAAGGATAACTCCCCGATCATTAAATCACC
**MR15**	TGCGAATTCCCCCTTGATGAAGATGAAGCATCATTTGTGGC
**MR16**	GTACTGCATGAAGCGTTAGCAATTTTCGAATAAGCACAA
**MR17**	GTAGGATCCGAAGCGTTAGCAATTTTCGAATAAGCACAA
**MR18**	CACCATGGGTAGTACAGCTAAAACGCTTAC
**MR19**	CACCATGGGTAATGGGACTGATATTAAGGCTAAG
**MR20**	TTAATACCAGCCGTTAGTTTCG
**MR21**	CTACACTTCCACATCCCTGAC

Table S2. Small Angle X-ray Scattering structural and molecular parameters

Distance Distribution	Rg(Å)	Dmax(Å)	Estimated MW
**HipB**	22.812 ± 0.136	79.8	26.0kDa
**HipB:DNA**	26.013 ± 0.349	88	38.6kDa
**HipAB**	37.133 ± 0.846	128	132.8kDa
**HipAB:DNA**	38.584 ± 0.601	132	146.3kDa

Table S3. Summary of thermodynamic parameters obtained by ITC.

Titration cell	Conc. μM	Syringe	Conc. μM	K_D nM	N	ΔH cal mol^{-1}	ΔS cal $mol^{-1}K^{-1}$
**HipA**	7	**HipB**	41	490	0.52	-3.429E4	-86.1
**DNA (26bp)**	6.8	**HipB**	63.5	290	1.13	1.381E4	76.2
**HipA-D306Q**	18.5	**HipB:DNA**	150.6	300	0.46	-3.167E4	-76.5
**DNA (76bp)**	2	**HipB**	50	230	2.12	1.308E4	74.2
**HipA**	13	**HipB Nt truncated**	103	420	0.44	-3.60E4	-91.5
